# Supplementary material for: In Vitro Antimicrobial Activity of Contezolid Against Mycobacterium tuberculosis and Absence of Cross-Resistance with Linezolid
Source: Microorganisms. 2025 Sep 22;13(9):2216. doi: 10.3390/microorganisms13092216 (PMC12472310; doi:10.3390/microorganisms13092216)
Supplement: Supplementary file 1 [file microorganisms-13-02216-s001.zip › microorganisms-3843724-supplementary.pdf]

Table S1. Whole-genome sequencing analysis of 9 strains of *M. tuberculosis*

Original strains	Strains ID	Mutations	
		Coding sequence	Gene
Y23	L43-Y23	*Rv0095c* D 57 E	*Rv0095c* C 171 G
		Rv0197 Y 749 *	*Rv0197* T 2247 G
		rplC C 154 R	*rplC* T 460 C
		Rv1148c L 297 L	*Rv1148c* T 889 C
		Rv1148c F 296 L	*Rv1148c* T 888 A
		Rv1148c D 294 D	*Rv1148c* T 882 C
		Rv1148c N 292 N	*Rv1148c* T 876 C
		Rv1148c N 292 H	*Rv1148c* A 874 C
		Rv1148c R 291 R	*Rv1148c* C 873 A
		Rv1148c A 289 A	*Rv1148c* G 867 C
		Rv1148c Q 288 Q	*Rv1148c* G 864 A
		lppB N 93 T	*lppB* A 278 C
		lppB D 94 E	*lppB* C 282 A
		lppB E 133 E	*lppB* A 399 G
		lppB I 137 V	*lppB* A 409 G
		lppB A 138 V	*lppB* C 413 T
		lppB A 139 T	*lppB* G 415 A
		lppB R 143 R	*lppB* A 429 G
		Rv2666 T 9 A	*Rv2666* A 25 G
		-	integrated_mobile_genetic_element 150 G contain
		ceoB G 75 C	*ceoB* G 223 T
	C39-Y23	*Rv0095c* D 57 E	*Rv0095c* C 171 G
		Rv1148c R 291 R	*Rv1148c* C 873 A
		Rv1148c A 289 A	*Rv1148c* G 867 C
		Rv1148c Q 288 Q	*Rv1148c* G 864 A
		dsbF I 7 V	*dsbF* A 19 G
		mce3R E 280 K	*mce3R* G 838 A
		lppB G 47 D	*lppB* G 140 A
		lppB E 133 E	*lppB* A 399 G
		lppB I 137 V	*lppB* A 409 G
		lppB A 138 V	*lppB* C 413 T
		lppB A 139 T	*lppB* G 415 A
		lppB R 143 R	*lppB* A 429 G
		Rv2666 T 9 A	*Rv2666* A 25 G
		-	integrated_mobile_genetic_element 150 G contain
	C41-Y23	*Rv0090* G 229 G	*Rv0090* T 687 C
		Rv0095c D 57 E	*Rv0095c* C 171 G
		Rv1371 H 95 Q	*Rv1371* C 285 A
		aroG L 191 L	*aroG* G 573 A
		lppB N 93 T	*lppB* A 278 C

		lppB D 94 E<br>*lppB* E 133 E<br>*lppB* I 137 V<br>*lppB* A 138 V<br>*lppB* A 139 T<br>*lppB* R 143 R<br>*Rv2666* T 9 A<br>-<br>*ceoB* G 75 C	*lppB* C 282 A<br>*lppB* A 399 G<br>*lppB* A 409 G<br>*lppB* C 413 T<br>*lppB* G 415 A<br>*lppB* A 429 G<br>*Rv2666* A 25 G<br>integrated_mobile_genetic_element 150 G contain<br>*ceoB* G 223 T
Y26	C14-Y26	*pks5* P 1755 T<br>*mas* V 496 G<br>*Rv2971* H 172 H<br>*Rv3786c* A 293 V	*pks5* C 5263 A<br>*mas* T 1487 G<br>*Rv2971* C 516 T<br>*Rv3786c* C 878 T
	C12-Y26	*mce3R* G 169 D<br>*mas* V 496 G	*mce3R* G 506 A<br>*mas* T 1487 G
	L4-Y26	*rplC* C 154 R	*rplC* T 460 C
Y117	C33-Y117	*esxM* L 96 L<br>*mce3R* R 272 H<br>*Rv2082* P 271 P	*esxM* G 288 C<br>*mce3R* G 815 A<br>*Rv2082* A 813 G
	C31-Y117	*esxM* L 96 L<br>*mce3R* G 245 D<br>*Rv2082* S 247 T<br>*Rv2082* S 247 S<br>*Rv2082* P 271 P	*esxM* G 288 C<br>*mce3R* G 734 A<br>*Rv2082* G 740 C<br>*Rv2082* T 741 C<br>*Rv2082* A 813 G
	L28-Y117	-<br>*Rv2082* S 247 T<br>*Rv2082* S 247 S	*rrl* G 2814 T<br>*Rv2082* G 740 C<br>*Rv2082* T 741 C

Table S2. Unreported mutations detected by whole genome sequencing in the strains induced by contezolid or linezolid

Mutants	Gene mutations referenced original strain (unreported)
L43-Y23	*Rv0095c, Rv1148c, lppB, Rv2666*
C39-Y23	*Rv0095c, Rv1148c, lppB, Rv2666*
C41-Y23	*Rv1371, Rv0090, Rv0095c, lppB, Rv2666*
L4-Y26	
C12-Y26	
C14-Y26	*Rv3786c*
L28-Y117	
C31-Y117	
C33-Y117	
